# PP05 Comparing Generative Artificial Intelligence And Expert-Driven Insights For Breast Cancer Chemotherapy Decisions: Implications For Health Technology Assessment Processes

**DOI:** 10.1017/S0266462325102250

**Published:** 2025-12-29

**Authors:** Bruno Barros, Carlos Alberto Magliano

## Abstract

**Introduction:**

Generative artificial intelligence (AI) is revolutionizing real-world evidence generation in health care. This study compared chemotherapy recommendations for women with breast cancer across different clinical risk profiles and Oncotype DX Breast Recurrence Score® test results, as obtained from a Delphi panel of experts, with recommendations generated by ChatGPT. The objective was to analyze concordances and differences between AI-generated and expert-driven insights.

**Methods:**

An online survey of 10 independent breast cancer experts, blinded to each other’s responses, assessed chemotherapy recommendations for patients with early stage, node-negative breast cancer. Responses were analyzed by clinical risk (high versus low), age group (≤50 versus >50 years), and recurrence score (RS) (<11, 11 to 25, >25) from Oncotype DX. ChatGPT, using automated prompt engineering, addressed the same scenarios as agent-based oncologists. Expert recommendations were summarized as arithmetic means, while ChatGPT responses were analyzed for concordance. A one-sample t-test compared mean estimates between the Delphi panel and ChatGPT results, highlighting differences in recommendations across groups.

**Results:**

The Delphi panel and ChatGPT provided clinically similar chemotherapy recommendations for patients evaluated with Oncotype DX, with no statistically significant differences in eight out of 12 scenarios. Both agreed on zero percent chemotherapy for patients with low clinical risk (RS<11) and showed comparable results for high clinical risk (RS>25), including patients under 50 years (8% versus 10%) and over 50 years (6% versus 5%). The largest divergence, which was statistically significant, was observed for patients with low clinical risk (RS 11 to 25) who were over 50 years (1% versus 20%). High-risk patients consistently received strong recommendations, with near perfect agreement for those with high clinical risk (RS>25) who were under 50 years (98% versus 95%).

**Conclusions:**

From a decision-making perspective, the responses from ChatGPT and the Delphi panel were very similar, suggesting that AI can effectively support the health technology assessment process. This alignment highlights AI’s potential to accelerate decision-making, offering a faster alternative to the traditional, time-consuming Delphi model while maintaining reliable chemotherapy recommendations.

